# Measuring visual search and distraction in immersive virtual reality

**DOI:** 10.1098/rsos.172331

**Published:** 2018-05-02

**Authors:** Bettina Olk, Alina Dinu, David J. Zielinski, Regis Kopper

**Affiliations:** 1 Jacobs University Bremen, Campus Ring 1, 28759 Bremen, Germany; 2 HSD University of Applied Sciences, Waidmarkt 3 and 9, 50676 Cologne, Germany; 3 Department of Systems Neuroscience, University Medical Center Hamburg-Eppendorf, Martinistrasse 52, 20251 Hamburg, Germany; 4 Duke University, Pratt School of Engineering, FCiemas Building, 101 Science Dr., Durham, NC 27708-0271, USA

**Keywords:** attention, control, virtual reality, flanker, ecological validity

## Abstract

An important issue of psychological research is how experiments conducted in the laboratory or theories based on such experiments relate to human performance in daily life. Immersive virtual reality (VR) allows control over stimuli and conditions at increased ecological validity. The goal of the present study was to accomplish a transfer of traditional paradigms that assess attention and distraction to immersive VR. To further increase ecological validity we explored attentional effects with daily objects as stimuli instead of simple letters. Participants searched for a target among distractors on the countertop of a virtual kitchen. Target–distractor discriminability was varied and the displays were accompanied by a peripheral flanker that was congruent or incongruent to the target. Reaction time was slower when target–distractor discriminability was low and when flankers were incongruent. The results were replicated in a second experiment in which stimuli were presented on a computer screen in two dimensions. The study demonstrates the successful translation of traditional paradigms and manipulations into immersive VR and lays a foundation for future research on attention and distraction in VR. Further, we provide an outline for future studies that should use features of VR that are not available in traditional laboratory research.

## 1. Introduction

Attentional resources are limited. We experience this each time we are distracted and unable to focus. Understanding the mechanisms that underlie distraction is of interest to cognitive psychology but also many applied disciplines, including engineering, software development and therapeutic interventions, to name a few. Seminal research has shown that the detection of targets among distractors and the efficiency of ignoring distracting items are affected by features that stimuli share (e.g. [1,2]). To assess target selection and distraction, participants may be asked to determine which one of two targets is present in a search array. For instance, in one condition participants may search for an N or X among the letters O. In another condition participants may search for an N or X among different angular letters, e.g. K, M, H, W and Z. Typically, reaction time (RT) is significantly longer in the latter condition, indicating higher demands on attention because the targets and distractors share features (e.g. [3]). To further assess distraction, the array may be flanked by a task-irrelevant stimulus that can be the same as the current target (congruent) or as the other possible target (incongruent). The usual result in a flanker paradigm [4] is that although participants should ignore the flanker, they respond faster when target and flanker are congruent rather than incongruent. This flanker effect indicates that the flanker received attention and that response competition occurred. Some studies have shown that flanker effects are smaller or absent when the demands that the search task places on attention are high (see [5–7]; but also see [8]). It has been proposed that in a condition in which finding a target is demanding and perceptual load is high, the attentional capacity allocated to the perception of stimuli is exhausted and the perception of the flanker is prevented [5]. However, when searching for an N or X among Os, finding a target among distractors is not demanding and perceptual load is said to be low and spare attentional capacity ‘leaks’ to the flanker, resulting in attention to and perception of the flanker.

An important issue critical to all psychological research is how experiments conducted in the laboratory and theories based on such experiments relate to human performance in daily life. After all, it is ‘daily’ human cognition and performance that psychologists wish to understand and explain. Ecological validity is a timely topic (e.g. [9–11]) and has been raised in the investigation of target selection and distraction. For example, individual differences in daily life distractibility have been considered [12]. Participants searched for a target letter (N or X) among distractor letters under low or high perceptual load (small Os or different angular letters, respectively). Flankers (N or X) were presented. Additionally, participants filled out the Cognitive Failures Questionnaire (CFQ) [13]. High CFQ scores that reflect high distractibility in daily life were associated with larger flanker effects (i.e. larger distraction) under low but not high load. This finding may suggest that an increase of perceptual load may be beneficial in daily tasks, particularly for persons who are easily distracted.

It is important to consider that most previous research in this domain used computerized displays together with letter search tasks. Simple stimuli such as letters are widely used in experimental psychology and many important theories have been established using this type of stimuli (e.g. [14,15]). An advantage of using simple stimuli is that they can be controlled well, e.g. their similarity can be manipulated systematically [16]. A disadvantage is that such stimuli lack ecological validity. Ideally, for maximally ecologically valid assessments participants should be tested in everyday situations. However, drawbacks would be difficulties in controlling attention-relevant aspects of daily life and manipulating the stimuli and conditions (but see [17]). Virtual reality (VR) allows building a bridge between traditional research in the laboratory and daily situations. In VR life-sized stimuli can be displayed in realistic settings without losing the advantage of controlling conditions. In particular investigations in *immersive* VR are beneficial because the sense of ‘presence’ within the environment is increased compared to other virtual environment technologies [18]. Although immersive VR has been used successfully in attention research (e.g. [19,20]) studies are rare. For instance, none of the previous VR studies has examined the impact of target–distractor discriminability on distraction and response competition. As the evident strength of VR is the possibility to replicate and alter real life situations, future studies should focus on this asset [21]. We therefore tested distraction in immersive VR using daily objects as stimuli. Our goals were to accomplish a transfer of traditional paradigms used for assessing attention and distraction to an immersive VR environment and to explore the attentional effects when daily objects are being used as stimuli. Importantly, we consider our study a first step in the direction of investigating distraction and response competition in a VR environment on which future studies can be based. Because the present study is the first that looks at distraction and response competition in VR, we used VR for the visual presentation of stimuli. We would like to emphasize though that further studies should take advantage of the more ecologically valid context that VR offers for studying cognition and the options that are available in VR but not traditional settings such as the effects of acting on objects in an immersive environment. However, to take the required first step, we consider it important to ensure contact of our experiment with previous studies on the one hand, e.g. by arranging stimuli in a similar way to previous studies and by asking for simple button presses, while increasing ecological validity by assessing distraction in immersive VR using daily objects on the other hand. In order to compare the results obtained in the VR environment to assessments in a ‘typical’ laboratory setting, we carried out a second experiment with two-dimensional (2D) presentation. In both experiments participants searched for daily items on the countertop of a virtual kitchen while instructed to ignore flanker items.

Although experiments in which images are presented in an immersive three-dimensional (3D) environment differ in various aspects from experiments in which stimuli are presented on a computer monitor in 2D [22], we expected that the pattern of results obtained with the two assessment methods should be similar. Previous studies comparing performance in 2D and 3D did not report marked differences. For instance, one study [20] revealed significant correlations between the results obtained with presentation on a CRT monitor and presentation via Oculus Rift when assessing conscious perception and visual working memory and test–retest reliability of the cognitive measurements. Also studies using immersive VR assessing scene memory and eye movements report results in agreement with 2D paradigms [22,23]. One particular study though [24] observed faster and more accurate visual search performance in a VR environment than in a 2D condition, suggesting performance in VR could be better than in a 2D condition. As in our study we only varied the presentation method and the environment but the stimuli and task remained the same and participants were not required to move or to interact with the stimuli in the VR environment, we would predict a similar pattern of results for both methods, with possibly faster responses in VR.

In our second experiment (Experiment 2), the 2D presentation was done on a standard CRT monitor in the laboratory. This provided us with the additional opportunity to track the eye movements of our participants. Eye tracking is a very sensitive method to measure the allocation of overt attention and can reveal attentional effects that are not apparent by only measuring RT and accuracy. Therefore, we decided to use this method in addition to manual RT measurements in Experiment 2 to verify typical search effects with our novel stimuli. Overall, we expected that participants should make more fixations until they reach the target when target discrimination is harder than easier, reflecting the more difficult and demanding visual search process with low target–distractor discriminability. Further, the assessment of fixations on the flanker was of interest to investigate ‘overt’ distraction, i.e. whether participants would look at the flanker at all or whether the flanker effect is rather mediated by covert attention. We also assessed whether target–distractor discriminability would modulate how often, when and for how long participants would look at the flanker.

## 2. Experiment 1

**Figure 1.**
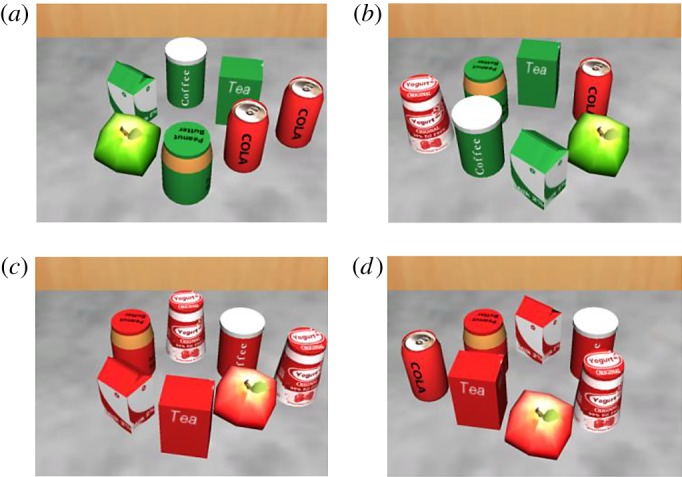
Example displays for (*a*) high discriminability, congruent (soda target, soda flanker), (*b*) high discriminability, incongruent (soda target, yoghurt flanker), (*c*) low discriminability, congruent (yoghurt target, yoghurt flanker), and (*d*) low discriminability, incongruent (yoghurt target, soda flanker).

Experiment 1 was carried out in immersive VR. The design of the experiment followed typical experiments for the investigation of distraction, using the flanker paradigm. Participants were situated in a full virtual kitchen, looking at one of the countertops, located in front of them. They had to indicate by button press on a handheld device which one of two target stimuli (red/white yoghurt or red soda can) was present in a circular array consisting of six stimuli. Target–distractor discriminability was varied. In one condition targets and distractors were shown in a different colour, e.g. the targets were red or red/white and distractors were presented in green and green/white, and thereby easy to discriminate from each other. In another condition, both targets and distractors were red and red/white, and thus shared colour and were difficult to discriminate. In accordance with previous research, we predicted that search should be harder and RT should be higher when targets and distractors share colour. Further, a flanker item—congruent or incongruent with the target—was presented to the left or right of the circular array (figure 1) and had to be ignored. RT should be higher when target and flanker are incongruent compared to congruent, resulting in a flanker effect. Additionally, the flanker effect may be larger when target–distractor discriminability is high (as perceptual load would be low) than when target–distractor discriminability is low (as perceptual load should be high).

### 2.1. Material and methods

#### 2.1.1. Participants

Twenty-two participants, recruited via e-mail at Duke University, gave informed consent, and filled out a survey, providing information on age (*M* = 34 years, s.d. = 13.8), gender (11 females), handedness (21 right-handed) and vision (11 corrected vision, none colour-blind). After testing, participants filled out the CFQ [13]. Scores ranged from 23 to 56 (*M* = 41.32, s.d. = 9.16). Participants received $8. The protocol was approved by the Institutional Review Board of Duke University.

#### 2.1.2. Apparatus

We used a six-sided active stereo CAVE-type system (Duke immersive Virtual Environment DiVE; http://virtualreality.duke.edu/). Head tracking with 6 degrees of freedom was provided by an Intersense IS-900 tracker. The hand controller was an Intersense IS-900 wand controller, used only for its button capability. Participants wore Crystal Eyes CE3 shutter glasses synced to projectors running at 110 Hz. An active-stereo alternated between left and right displayed frames, which led to an effective frame rate of 55 Hz for the user. A cluster of computers (1 computer per projector) plus one computer as a master computer powered the system. All computers had NVIDIA Quadro FX 5600G graphics cards. The Simulation was created in the Unity3D game engine and used the MiddleVR plugin to facilitate running in our CAVE-type system.

#### 2.1.3. Stimuli and procedure

The stimulus set consisted of 13 objects: a fixation stimulus (white cup), two target or flanker objects that were easy to discriminate from each other (red/white yoghurt, red soda can) and 10 distractor objects (red or green apple, red or green tea box, red/white or green/white coffee jar, red/white or green/white milk carton, red or green peanut butter jar). Colours were defined by the following RGB values: red = 191, 24, 24; white = 255, 255, 255; green = 29, 119, 47. The height of all objects was scaled to the height of the soda can (0.106 m; 5.52° of visual angle). The apple was slightly smaller (0.098 m; 5.10°) because a larger apple appeared unnatural. Width and depth varied slightly to keep objects looking natural (e.g. a milk carton of the same dimensions as a soda can appeared unnatural). Six stimuli (five distractors, one target) were arranged in a circular array (diameter 0.2 m). Distractor location was randomized. The target (soda or yoghurt, each presented in 50% of the trials) appeared equally often at each location. A flanker (soda or yoghurt, presented equally often) was displayed on the left or right of the circle (0.175 m distance between centre of the circle and flanker).

The experiment contained four conditions, between which target–flanker congruency and target–distractor discriminability were varied. On congruent trials target and flanker were the same; on incongruent trials target and flanker differed. On trials with high target–distractor discriminability targets and distractors were of different colour (targets red and red/white, distractors green and green/white), while on low target–distractor discriminability trials targets and distractors shared colour (both red and red/white). The experiment consisted of 192 trials, separated into two blocks. The conditions were randomized within a block. Participants completed 16 practice trials. Example displays are illustrated in figure 1.

Participants were shown example displays on paper to explain the task. To familiarize themselves with the VR environment, they went into the environment with the experimenter and were allowed to move and look around the entire virtual kitchen. During testing they stood on a pre-determined carpet tile in the centre of the VR environment, facing the countertop on which the stimuli appeared. The distance between eye level and location of the objects was approximately 1.1 m, varying slightly depending on the height of a participant.

A trial started with the presentation of an upright or upside down cup in the centre that had to be fixated at the beginning of each trial. After 1000 ms the stimulus array and flanker were shown for 2000 ms. Participants pressed one of two response buttons on the wand to indicate which target was presented in the circle. Half of the participants pressed the top button for soda and the bottom button for yoghurt, half of the participants vice versa. Pressing a button elicited a dart sound effect (i.e. the moment the dart is thrown into the board) to provide feedback that a button was pressed but not whether the response was correct or incorrect. Participants were instructed to ignore the flanker. The inter-trial interval was set at 2000 ms. Participants were offered to take a break, to take off the shutter glasses and leave the VR environment between blocks. The end of a block was indicated by the Microsoft windows ‘tada’ sound. After the experiment, participants filled out the CFQ. An entire testing session took 35–45 min.

### 2.2. Results

Mean RTs of correct response trials were calculated per condition. Trials with no (0.4%) or incorrect responses (1.8%) and outliers (1.5%, RT lower/higher than 2.5 s.d. than the mean per participant per condition) were discarded from the analysis. As error rate was very low, it was not analysed further.

#### 2.2.1. Effects of congruency and target–distractor discriminability

**Figure 2.**
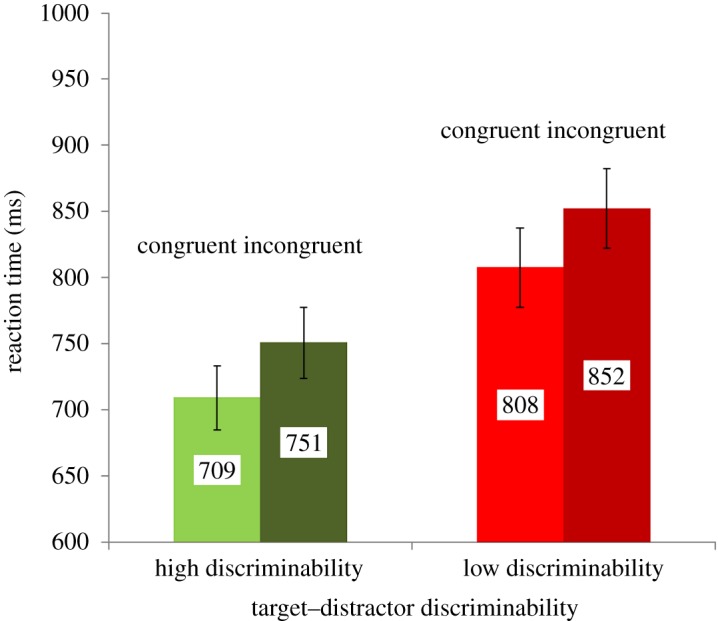
Reaction time as a function of congruency and target–distractor discriminability. Error bars represent standard errors of the mean.

A 2 (congruency: congruent versus incongruent) × 2 (discriminability: high versus low) analysis of variance (ANOVA) with repeated measures revealed that participants were slower on incongruent than congruent trials, *F*_1, 21_ = 37.96, *p* < 0.001, $\eta_{p}^{2}=0.644$. Further, they were slower when target–distractor discriminability was low compared to high, *F*_1, 21_ = 61.23, *p* < 0.001, $\eta_{p}^{2}=0.745$. The interaction between congruency and target–distractor discriminability did not reach significance, *F*_1, 21_ = 0.04, *p* = 0.845, $\eta_{p}^{2}=0.002$. The results are illustrated in figure 2.

#### 2.2.2. Exploration of associations between distraction, Cognitive Failures Questionnaire scores and age

We explored whether there is an association between the flanker effect and CFQ scores. High CFQ scores should reflect high distractibility in daily life. We determined the flanker effect (RT incongruent minus congruent) for high and low target–distractor discriminability trials per participant and calculated Pearson correlations. CFQ scores did not correlate with the flanker effect neither under high target–distractor discriminability, *r* = −0.302, *N* = 22, *p* = 0.172, two-tailed; nor under low discriminability, *r* = 0.037, *N* = 22, *p* = 0.871, two-tailed. We further divided participants into a high-CFQ (more distracted) and a low-CFQ (less distracted) group, using a median split of the CFQ scores (*Mdn* = 41). A 2 (congruency: congruent versus incongruent) × 2 (discriminability: high versus low) × 2 (CFQ-group: low, high) ANOVA with repeated measures confirmed the main effects of congruency, *F*_1, 20_ = 36.19, *p* < 0.001, $\eta_{p}^{2}=0.644$, and target–distractor discriminability, *F*_1, 20_ = 59.23, *p* < 0.001, $\eta_{p}^{2}=0.748$, but no other effects were significant, all *p* > 0.214. In our high target–distractor discriminability condition, if anything, the extent of the flanker effect was numerically *smaller* for participants with high CFQ scores (25 ms) than for participants with low CFQ scores (56 ms). Participants with low CFQ scores responded in general numerically faster, in particular when target and flanker were congruent in the high target–distractor discriminability condition, resulting in a large difference between congruent and incongruent trials.

We had not restricted the sample to the usual age group of young adults. Thus we were able to explore potential age effects. Indeed, age correlated positively with the flanker effect for high target–distractor discriminability trials, *r* = 0.444, *N* = 22, *p* = 0.039, two-tailed, but not for low target–distractor discriminability trials, *r* = 0.044, *N* = 22, *p* = 0.845, two-tailed.

#### 2.2.3. Effects of distance between participant and target location

To assess whether the distance between the observer and the target affected RT and distraction, we parsed the display into four areas (near: position nearest to the observer/at the bottom of the circular array (i.e. peanut butter jar in figure 1*a*), intermediate 1: position at the lower middle of the circular array (i.e. apple and soda can in figure 1*a*), intermediate 2: at the upper middle of the circular array (i.e. milk carton and tea box in figure 1*a*) and far: position furthest away from the observer/at the top of the circular array (i.e. coffee jar in figure 1*a*)). A 2 (congruency: congruent versus incongruent) × 4 (distance: near, intermediate 1, intermediate 2, far) ANOVA with repeated measures revealed, apart from a significant congruency effect, *F*_1, 21_ = 27.85, *p* < 0.001, $\eta_{p}^{2}=0.570$, that distance had an effect on RT, *F*_3, 63_ = 12.57, *p* < 0.001, $\eta_{p}^{2}=0.374$. When targets were located at the far position responses were faster than for all other distances (far (739 ms) versus intermediate 1 (770 ms), *t*_21_ = 2.61, *p* < 0.05; far versus intermediate 2 (810 ms), *t*_21_ = 5.64, *p* < 0.001; far versus near (783 ms), *t*_21_ = 2.92, *p* < 0.01). Responses were slowest to targets in the position intermediate 2 (intermediate 2 versus intermediate 1, *t*_21_ = 5.39, *p* < 0.001; intermediate 2 versus near, *t*_21_ = 2.23, *p* < 0.05). There was no difference between the intermediate 1 and the near distance, *t*_21_ = 1.26, *p* = 0.223. Further, an interaction between distance and congruency was observed, *F*_3, 63_ = 3.99, *p* < 0.05, $\eta_{p}^{2}=0.160$. Flanker effects were significantly larger for the far than for the near distance, *t*_21_ = 2.85, *p* < 0.05, for the intermediate 2 than for the near distance, *t*_21_ = 3.18, *p* < 0.005, and there was a trend for a larger effect for the intermediate 1 compared to the near distance, *t*_21_ = 2.02, *p* = 0.057.

#### 2.2.4. Effects of distance between target and flanker locations

To assess whether the distance between target and flanker affected RT and distraction, we defined three distances: close (when target and flanker were both located on the right side or both on the left side, i.e. as in figure 1*a*), intermediate (when the target was located at the top or bottom of the circular array, i.e. as in figure 1*c*) and far (when the target was located on the left side and the flanker on the right side and vice versa, i.e. as in figure 1*b*). A 2 (congruency: congruent versus incongruent) × 3 (distance: close, intermediate, far) ANOVA with repeated measures showed, apart from a congruency effect, *F*_1, 21_ = 37.4, *p* < 0.001, $\eta_{p}^{2}=0.640$, an effect of distance, *F*_2, 42_ = 8.51, *p* < 0.005, $\eta_{p}^{2}=0.288$. Participants responded significantly faster to targets when they were located at an intermediate distance (761 ms) to the flanker (i.e. at the top or bottom of the circular array) than far from the flanker (800 ms), *t*_21_ = 4.19, *p* < 0.001. The differences between close (780 ms) and far and between close and intermediate showed only a trend, *t*_21_ = 2.03, *p* = 0.055; *t*_21_ = 2.07, *p* = 0.052, respectively. There was no interaction between distance and congruency, *F*_2, 42_ = 1.24, *p* = 0.301, $\eta_{p}^{2}=0.056$.

### 2.3. Discussion

Experiment 1 assessed the effects of congruency and target–distractor discriminability in a flanker task presented in immersive VR, using daily objects as stimuli. Based on previous research in the laboratory we predicted effects of congruency, i.e. RT should be higher when target and flanker are incongruent compared to congruent, and of target–distractor discriminability, i.e. RT should be larger when target–distractor discriminability is low compared to high. Both effects were reflected in our data, showing that participants were unable to ignore the flanker and that search required indeed more time when targets and distractors shared colour. These results demonstrate that our novel stimuli and set-up in the immersive VR environment are suited to elicit the expected effects and that our goal to transfer a traditional paradigm into an ecologically more valid setting, using daily items, was reached. We did not observe an interaction between congruency and target–distractor discriminability. The flanker effect was comparable in both search conditions. Possible explanations may be that presentation duration was longer (2000 ms) than in previous studies that showed such an interaction (50 or 100 ms) [3], in light of a study that reported effects of stimulus duration on flanker effects under low and high perceptual load [25]. When in that study the entire stimulus array, containing target, distractors and flanker, was presented for a limited time, i.e. 100 ms, flanker effects were observed for low but not high load. However, when the entire stimulus array was presented and it was waited until a response was made, then flanker effects were present in both conditions. In our study, we presented the stimuli for a longer duration because in everyday life stimuli would also be present for longer. A longer presentation duration may have lifted encoding restrictions that could be important for an interaction to occur. Further, we mixed conditions of different target–distractor discriminability and did not run the conditions in separate blocks. The reason for this was as well that in everyday life we are confronted with high and low target–distractor discriminability ‘displays’ that vary frequently and are not ‘blocked’. It has been reported that when conditions were mixed, congruency effects were similar in both conditions, in line with our findings [8]. In addition, in our study the conjunction of features that define an object was more complex than typical conjunctions of features defining letters—the stimulus type that is traditionally used. Also, when everyday objects are presented, we are dealing with very familiar stimuli that activate semantic knowledge. Interestingly, no interaction has been reported when participants were very familiar with a flanker stimulus, e.g. a famous face [26], or when participants possessed expertise, e.g. when musical instrument flankers were presented to musicians [27]. It is possible that daily objects, which people are very familiar with, may be harder to ignore (see also [28]). Also, objects will activate semantic knowledge, which can guide visual attention (e.g. [29,30]) so that participants may not be processing and focusing solely on low-level properties such as colour in our experiment but also on higher-level properties. This additional focus may reduce the impact of perceptual aspects. While we acknowledge that disentangling potential effects of methodological variations is surely important, we would like to emphasize that they are of lesser importance to the outcome of our present study, since we focused on increasing ecological validity. After all, in everyday situations objects are even more complex than in our study, and are displayed in a more unpredictable sequence than two conditions in a randomized order and are presented for even longer. We are thus supporting the idea that future research should focus on approaching ‘real world’ situations even more than we did here and make use of assessment options of immersive VR that we did not yet use in this initial work.

We further explored associations between distraction, i.e. flanker effects, and CFQ scores since the latter should reflect distractibility in daily life. CFQ scores did not correlate with the flanker effect. This lack of a correlation between flanker effect and CFQ scores cannot simply be attributed to a potentially small sample size because the correlational analysis considering age yielded significant effects. Results of our analysis using a median split of CFQ scores showed that in the high target–distractor discriminability condition participants with high CFQ scores, who should be more distracted, showed a numerically smaller flanker effect, indicating less distraction in the experiment, than participants with low CFQ scores. The observation that participants with low CFQ scores seemed to respond faster, especially when targets and distractors are easier to discriminate and target and flanker were congruent, is of interest. Such faster responses may indicate a tendency for a superior ability of the low CFQ group to focus in the present task. Investigation of CFQ scores was not a central part of our study but we suggest that including this instrument in future studies would be of interest to elucidate potential associations between distraction and performance in an experimental set-up. Interestingly, with respect to age effects we observed that the older the participants were, the more distracted they were in the high target–distractor discriminability condition. This finding agrees with reports that older adults show larger distraction effects under low load than younger adults [31] and should encourage further investigations of age effects on attention and distraction also in VR.

The analysis of spatial effects showed that when targets were located at the far position in relation to the observer responses were faster than for all other distances and responses were slowest to targets in the position intermediate 2. This result may indicate that objects further away may be processed earlier. However, it is possible that the objects in the far location are also coded as being presented at the top of the display and may thus be processed earlier than other objects, in line with a visual search strategy that begins search at the top of a display, and may, in addition, be more salient than objects in the intermediate positions because there is only one far object present compared to multiple objects at the intermediate position. The finding that flanker effects were significantly larger when the target was located further away from the observer may also indicate that even if responses are faster, processing of the targets at the far location may be less meticulous, leading to more distraction than for targets presented nearby. With respect to the distance between target and flanker, participants responded the fastest to targets when they were located at an intermediate distance from the flanker and slowest when they were located far from the flanker. In line with the previous analysis targets located at the intermediate position are those of which some are also located far from the observer (at the top of the display) where they may be responded to faster. Thus, the coding of distance from the observer as well as distance between target and flanker may not be independent.

## 3. Experiment 2

Experiment 1 showed that typical effects of congruency and target–distractor discriminability were observed during testing in an immersive VR environment, which confirms that our materials and set-up were suitable to elicit the basic effects. For further assessing the potential of immersive VR for this line of research, it is important to address hypothetical effects of the testing environment and to investigate whether the effects observed in VR would be stable and similar to effects in a 2D environment using the same stimuli and task. In order to do so, we replicated the experiment in a more ‘typical’ laboratory situation, using a computer monitor. Also, as outlined above, testing in the laboratory in front of a CRT monitor enabled us to track eye movements of our participants and led to the addition of this measurement in Experiment 2. As outlined above, we expected more fixations until a target is reached when target discrimination is harder. Moreover, we assessed whether the flanker would be fixated and if so, how often, when and for how long.

### 3.1. Material and methods

#### 3.1.1. Participants

Twenty-six participants, recruited via e-mail at Jacobs University, gave informed consent, and filled out a survey, providing information on age (*M* = 21.95 years, s.d. = 1.95), gender (11 females), handedness (25 right-handed, 1 ambidextrous) and vision (6 corrected vision, none colour-blind). After testing, participants filled out the CFQ [13]. Scores ranged from 6 to 68 (*M* = 38.96, s.d. = 15.57). All participants were compensated with 5 euros. The experiment was conducted in accordance with the ethical standards laid down in the 1964 Declaration of Helsinki.

#### 3.1.2. Apparatus

Displays were presented on a computer with a 21^″^ CRT monitor (ViewSonic G220F). Experiment Builder software was used to run the experiment and eye movements were recorded with a head-mounted EyeLink II eye tracker (SR Research Canada). Testing was preceded by a 9-point grid calibration and validation for which participants were instructed to saccade to a black circle (0.5°) on a white background, which appeared sequentially and randomly at nine points in a square array. Eye movements were recorded at 500 Hz sample rate. Saccade onset was defined as a change in eye position with a minimum velocity of 30° s^−1^ or a minimum acceleration of 8000° s^−2^. Head movements were minimized using a chin rest.

#### 3.1.3. Stimuli and procedure

The stimulus set, conditions and procedure were the same as in Experiment 1 with the exception that testing did not take place in VR but in the laboratory in front of a computer monitor. Screenshots of the 3D displays of Experiment 1 were used. The size of the objects varied slightly depending on the position of an item in the circular array. For instance, when the soda can was shown on the left its height was 9.8 cm (5.61°), in the back 8.8 cm (5.04°), and in the front 10.2 cm (5.84°), thus on average (5.5°) of the same size as in Experiment 1. The diameter of the circular array was 23 cm and the distance between the centre of the circle and the flanker was about 17 cm. The distance between the eyes of the participants and the screen was 100 cm. The experiment consisted of 192 trials, separated into two blocks. The conditions were randomized within a block. Participants completed 16 practice trials and pressed one of two response buttons on a standard keyboard located in front of them to indicate which target was shown in the circle. Half of the participants pressed the ‘arrow up’ button for soda and the ‘arrow down’ button for yoghurt, half of the participants vice versa. Pressing a button elicited a dart sound effect to provide feedback that a response was made but not whether the response was correct or incorrect. Participants were instructed to ignore the flanker. They did not obtain any instructions with respect to eye movements. After the experiment, the participants filled out the CFQ. An entire testing session took 35–45 min.

### 3.2. Results

Trials on which the initial fixation was not in the centre (0.1%), fixations were off screen (1.4%), the response was incorrect (4%) and outliers (2.5%, RT lower/higher than 2.5 s.d. than the mean per participant per condition) were discarded from the analysis. As error rate was very low, it was not analysed further.

### 3.3. Manual reaction time

#### 3.3.1. Effects of congruency and target–distractor discriminability

**Figure 3.**
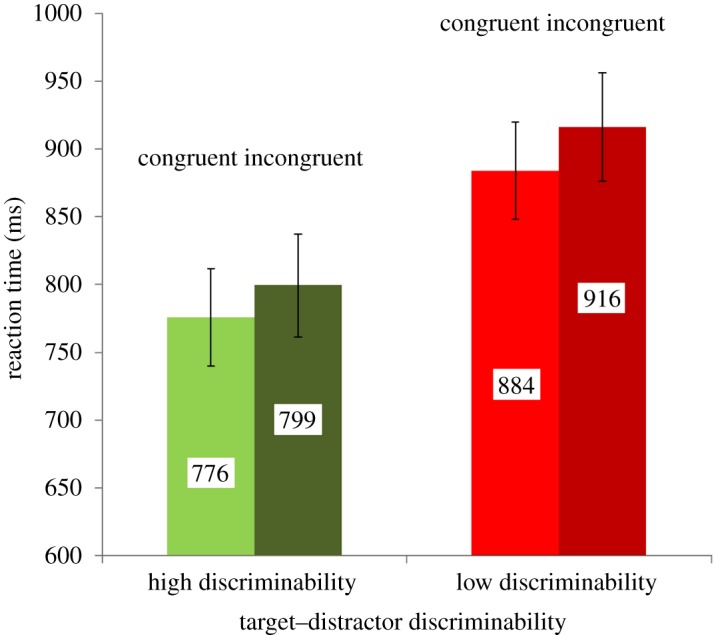
Manual reaction time as a function of congruency and target–distractor discriminability. Error bars represent standard errors of the mean.

Mean response times of correct response trials were calculated per condition. The results are depicted in figure 3. A 2 (congruency: congruent versus incongruent) × 2 (discriminability: high versus low) ANOVA with repeated measures revealed that participants were slower on incongruent than congruent trials, *F*_1, 25_ = 12.56, *p* < 0.005, $\eta_{p}^{2}=0.334$. Further, they were slower when target–distractor discriminability was low, *F*_1, 25_ = 91.4, *p* < 0.001, $\eta_{p}^{2}=0.785$. The interaction between both factors was not significant, *F*_1, 25_ = 0.373, *p* = 0.547, $\eta_{p}^{2}=0.015$.

#### 3.3.2. Comparison between Experiment 1 and 2

In order to compare the effects between both experiments, a 2 (experiment: E1 versus E2) × 2 (congruency: congruent versus incongruent) × 2 (discriminability: high versus low) ANOVA with repeated measures was carried out. Congruency, *F*_1, 46_ = 44.03, *p* < 0.001, $\eta_{p}^{2}=0.489$, and discriminability effects, *F*_1, 46_ = 149.62, *p* < 0.001, $\eta_{p}^{2}=0.765$, were replicated. Again, there was no interaction between the two factors, *F*_1, 46_ = 0.314, *p* = 0.578, $\eta_{p}^{2}=0.007$. There was no main effect of experiment and no interaction with the factor experiment, all *p* > 0.181.

#### 3.3.3. Exploration of associations between distraction and Cognitive Failures Questionnaire scores

We explored whether there was an association between the flanker effect and CFQ scores. We determined the flanker effect (RT incongruent minus congruent) for high and low target–distractor discriminability trials per participant and calculated Pearson correlations. As the CFQ was not assessed for two participants, we used the data of the remaining 24 participants for the analyses. CFQ score neither correlated with the flanker effect with high target–distractor discriminability, *r* = −0.237, *N* = 24, *p* = 0.264, two-tailed; nor with low target–distractor discriminability, *r* = 0.267, *N* = 22, *p* = 0.204, two-tailed. We further divided participants into a high-CFQ (more distracted) and a low-CFQ (less distracted) group, using a median split of the CFQ scores (*Mdn* = 38.5). A 2 (congruency: congruent versus incongruent) × 2 (discriminability: high versus low) × 2 (CFQ-group: low, high) ANOVA with repeated measures confirmed the main effects of congruency, *F*_1, 22_ = 12.36, *p* < 0.005, $\eta_{p}^{2}=0.360$, and target–distractor discriminability, *F*_1, 22_ = 84.10*, p* < 0.001, $\eta_{p}^{2}=0.793$, but no other effects were significant, all *p* > 0.178. In the high discriminability condition the extent of the flanker effect was numerically *smaller* for participants with high CFQ scores (22 ms) than for participants with low CFQ scores (32 ms). Participants with low CFQ scores responded in general numerically faster, especially when target and flanker were congruent in the high target–distractor discriminability condition.

#### 3.3.4. Effects of distance between participant and target location

To assess whether the distance between the observer and the target affected RT and distraction, we again parsed the display into four areas as in Experiment 1. A 2 (congruency: congruent versus incongruent) × 4 (distance: near, intermediate 1, intermediate 2, far) ANOVA with repeated measures revealed, apart from a significant congruency effect, *F*_1, 25_ = 13.18, *p* < 0.005, $\eta_{p}^{2}=0.345$, that the distance had an effect on RT, *F*_3, 75_ = 15.48, *p* < 0.001, $\eta_{p}^{2}=0.382$. When targets were located far (797 ms), responses were faster than for all other distances (far versus intermediate 1 (865 ms), *t*_25_ = 5.64, *p* < 0.001; far versus intermediate 2 (851 ms), *t*_25_ = 5.14, *p* < 0.001; far versus near (830 ms), *t*_25_ = 3.16, *p* < 0.005). Responses were slower to targets for the distance intermediate 1 than near, *t*_25_ = 3.18, *p* < 0.005, and there was a trend for the comparison between intermediate 2 versus near, *t*_25_ = 1.91, *p* = 0.067. There was no significant difference between the two intermediate distances, *t*_25_ = 1.64, *p* = 0.114. Further, an interaction between distance and congruency was observed, *F*_3, 75_ = 18.03, *p* < 0.001, $\eta_{p}^{2}=0.419$. Flanker effects were significantly larger for the far than the near position, *t*_25_ = 5.06, *p* < 0.001, the far than the intermediate 1 position, *t*_25_ = 4.65, *p* < 0.001; and numerically larger for the far compared to the intermediate 2 position, *t*_25_ = 2.05, *p* = 0.051. Further, flanker effects were larger for the intermediate 1 than for the intermediate 2 position, *t*_25_ = 2.44, *p* < 0.05; for the intermediate 1 than for the near position, *t*_25_ = 3.58, *p* < 0.005; and the intermediate 2 than the near position, *t*_25_ = 5.03, *p* < 0.001.

#### 3.3.5. Effects of distance between target and flanker locations

The data were analysed analogous to Experiment 1. A 2 (congruency: congruent versus incongruent) × 3 (distance: close, intermediate, far) ANOVA with repeated measures showed, apart from a congruency effect, *F*_1, 25_ = 11.03, *p* < 0.005, $\eta_{p}^{2}=0.306$, an effect of distance, *F*_2, 50_ = 45.7, *p* < 0.001, $\eta_{p}^{2}=0.646$. Participants responded significantly faster to targets when they were located at an intermediate distance to the flanker (815 ms) than close (896 ms) to the flanker, *t*_25_ = 7.81, *p* < 0.001, and when located far (821 ms) compared to close, *t*_25_ = 8.41, *p* < 0.001. There was an interaction between distance and congruency, *F*_2, 50_ = 3.3, *p* < 0.05, $\eta_{p}^{2}=0.117$. The flanker effect was larger when targets were located far compared to the intermediate distance, *t*_25_ = 2.26, *p* < 0.05. There was a trend for a larger flanker effect for the far compared to the close distance, *t*_25_ = 1.99, *p* = 0.057.

### 3.4. Eye movements

#### 3.4.1. Fixations on the target

For the analyses of fixations, the display screen was parsed into areas of interest including one of the objects each. The analysis showed that in 70.9% of the trials the area of interest of the target was fixated at least once per trial. However, two participants maintained central fixation throughout the experiment. When those two were removed from the fixation analyses, 76.9% of the trials contained fixations on the target.

Analogous to the manual RT data, we carried out a 2 (congruency: congruent versus incongruent) × 2 (discriminability: high versus low) ANOVA with repeated measures to analyse the average number of fixations that were made before participants looked at the target. The statistical results of this analysis are reported in table 1. More search moves (fixations on areas of interest other than the target) occurred until the target was looked at when target–distractor discriminability was low compared to high. There was no effect of congruency on the number of fixations until the target was reached and no significant interaction. A further analysis showed that the number of fixations until the target was looked at was affected by the distance of the observer to the target, *F*_3, 69_ = 22.35, *p* < 0.001, $\eta_{p}^{2}=0.493$, and discriminability, *F*_1, 23_ = 39.5, *p* < 0.001, $\eta_{p}^{2}=0.632$. Both factors interacted, *F*_3, 69_ = 6.39, *p* < 0.005, $\eta_{p}^{2}=0.217$. The number of fixations until the target was fixated was highest for the intermediate 2 position, in particular when discriminability was low (4.9) compared to high (4.0), *t*_23_ = 4.85, *p* < 0.001, although there were also significant differences for the intermediate 1 (low: 4.0, high: 3.7, *t*_23_ = 3.44, *p* < 0.005), and the near (low: 4.0, high: 3.4, *t*_23_ = 4.09, *p* < 0.001) positions as well, and a trend for the far position (low: 3.2, high: 3.0), *t*_23_ = 1.82, *p* = 0.082. An additional analysis showed that, apart from discriminability, *F*_1, 23_ = 40.91, *p* < 0.001, $\eta_{p}^{2}=0.640$, the distance between target and flanker also affected responses, *F*_2, 46_ = 18.26, *p* < 0.005, $\eta_{p}^{2}=0.442$. Participants made fewer fixations until they looked at the target when the target was located at the intermediate distance from the flanker (3.4) compared to the close distance (4.0), *t*_23_ = 4.04, *p* < 0.005, or far distance (4.0), *t*_23_ = 7.69, *p* < 0.001. For this latter analysis there was no interaction between distance and discriminability, *F*_2, 46_ = 0.054, *p* = 0.947, $\eta_{p}^{2}=0.002$.

**Table 1.** Mean and standard deviations of the average number of fixations until fixation on the target and on the flanker, percentage of trials with fixations on the flanker and duration of fixations on the flanker.

	low target–distractor discriminability		high target–distractor discriminability		
	congruent	incongruent	congruent	incongruent	results of ANOVA
average number of fixations until fixation on the target	*M* *=* 3.9; s.d. = 0.84	*M* *=* 4.2; s.d. = 0.88	*M* *=* 3.6; s.d. = 0.80	*M* *=* 3.6; s.d. = 0.78	***discriminability: F*_**1, 23**_ = 32.89, *p* < 0.001,** $\eta_{\text{p}}^{\text{2}}=\text{0.588}$ *congruency: F*_1, 23_ = 1.74, *p* = 0.200, $\eta_{p}^{2}=0.070$ *discriminability × congruency*: *F*_**1, 23**_ = 1.0, *p* = 0.326, $\eta_{p}^{2}=0.042$
percentage of trials with fixations on the flanker	*M* = 10.35%; s.d. = 18.9%	*M* = 11.95%; s.d. = 0.21%	*M* = 10.94%; s.d. = 8.86%	*M* = 13.15%; s.d. = 0.92%	*discriminability: F*_1, 25_ = 0.641, *p* = 0.431, $\eta_{p}^{2}=0.025$ *congruency*: *F*_1, 25_ = 2.79, *p* = 0.107, $\eta_{p}^{2}=0.101$ *discriminability × congruency*: *F*_1, 25_ = 0.125, *p* = 0.727, $\eta_{p}^{2}=0.005$
average number of fixations until fixation on the flanker^a^	*M* = 6.9; s.d. = 1.4	*M* = 6.7; s.d. = 1.5	*M* = 5.6; s.d. = 1.3	*M* = 5.2; s.d. = 1.2	***discriminability: F*_**1, 13**_ = 44.72, *p* < 0.001,** $\eta_{\text{p}}^{\text{2}}=\text{0.775}$ *congruency*: *F*_1, 13_ = 0.94, *p* = 0.349, $\eta_{p}^{2}=0.068$ *discriminability × congruency*: *F*_1, 13_ = 0.05, *p* = 0.826, $\eta_{p}^{2}=0.004$
duration of fixation on the flanker^a^	*M* = 225 ms; s.d. = 199 ms	*M* = 249 ms; s.d. = 131 ms	*M* = 256 ms; s.d. = 101 ms	*M* = 288 ms; s.d. = 102 ms	*discriminability*: *F*_1, 13_ = 3.703, *p* = 0.106, $\eta_{p}^{2}=0.189$ *congruency: F*_1, 13_ = 0.035, *p* = 0.855, $\eta_{p}^{2}=0.033$ *discriminability × congruency*: *F*_1, 13_ = 0.151, *p* = 0.704, $\eta_{p}^{2}=0.011$

^a^Analysis based on the data of the 14 participants who showed fixations on the flanker in each combination of the factors discriminability and congruency; *M* = mean; s.d. = standard deviations of the mean; significant effects are marked in bold font.

#### 3.4.2. Fixations on the flanker

We conducted three separate 2 (congruency: congruent versus incongruent) × 2 (discriminability: high versus low) ANOVAs with repeated measures to analyse (i) whether participants looked at the flanker, (ii) if so, with which fixation and (iii) for how long. The results of the three analyses are reported in table 1.

The first analysis showed that overall, participants looked at the flanker only rarely, i.e. in 11.5% of all valid trials with a correct response. The percentage of trials in which participants looked at the flanker was modulated neither by target–distractor discriminability nor by congruency and the factors did not interact. The second and third analyses were, due to the rare percentage of trials with fixations on the flanker, based on the data of 14 participants only who showed fixations on the flanker in each combination of the factors target–distractor discriminability and congruency. The second analysis showed that participants looked at the flanker earlier when target–distractor discriminability was high compared to low. The earlier fixation of the flanker for high compared to low target–distractor discriminability was not modulated by congruency. There was neither a main effect of congruency nor an interaction. The third analysis^1^ on the duration of the fixations on the flanker did not return any significant effects.

### 3.5. Discussion

The manual RT data replicated the results of Experiment 1. Flanker effects were observed, i.e. participants responded more slowly when flankers were incongruent compared to congruent. This result indicates that flankers could not be ignored and that a conflict occurred. Further, effects of target–distractor discriminability between targets and distractors occurred. Participants responded slower in lower compared to higher target–distractor discriminability trials. Similar to Experiment 1, no interaction between the two factors occurred. The analyses of correlations between flanker effects and CFQ scores also replicated the results of Experiment 1. This outcome is important because it demonstrates that our novel materials and the procedure elicit reliable effects. The spatial analyses showed that when targets were located far from the observer responses were faster than for all other distances and the flanker effect was largest for the far location. Also, participants responded significantly faster to targets when they were located at an intermediate distance to the flanker, i.e. at the top or bottom of the circular array, a result similar to Experiment 1 but were now faster to respond to targets far from the flanker than close. Experiment 2 showed an interaction between distance and congruency, i.e. there was no significant flanker effect in Experiment 2 when target and flanker were located close by, which was not apparent in Experiment 1. This result suggests that there can be subtle differences between assessments in 2D and 3D that justify further investigation.

Taken together, the results of Experiments 1 and 2 were largely the same, in particular regarding effects of congruency and target–distractor discriminability. In our view, this fact does not undermine the usefulness of assessing attention and distraction in immersive VR, i.e. assuming that testing in VR would not be necessary because the same effects can be observed in 2D. To us, the results show that the materials and procedure are ‘safe’ to use also in VR, i.e. that the VR environment does not have any side effects that may undermine the effects. Our findings are in line with previous studies that also reported similar results in 2D and 3D. We used VR as a medium for visual presentation of stimuli and the next important steps would be to include further options that VR has to offer that are not available when testing with 2D presentations in front of a computer screen such as interaction with the environment. It is well possible that differences in results would occur under such conditions. Previous work has shown that, for example, exposure-related experience with the environment has larger effects in 3D than 2D and that memory may be used more in 3D than 2D [22].

We included eye tracking in Experiment 2 and overall, we expected that participants should make more fixations until they reach the target when target–distractor discriminability is low compared to high. The analyses of the trials on which the target area of interest was looked at confirmed this prediction. The analysis of fixations on the target further showed that it was in fact possible to determine the target and complete the task without eye movements to the target but that most participants chose to move their eyes and to also look at the target in the majority of the trials. Further, the spatial analyses showed that the target was looked at earlier for the far than the intermediate 2 position when distance between observer and target was considered, partly in line with the RT results where RT was fastest for the far distance. Considering the distance between target and flanker, the RT analyses had shown the fastest RT for targets at an intermediate distance from the flanker. This result is underlined by the eye movement patterns as fewer fixations were made until the target was reached when the distance between target and flanker was intermediate.

Regarding the assessment of fixations on the flanker, we asked whether the flanker would be looked at, i.e. whether distraction would be ‘overt’ and whether target–distractor discriminability would impact how often, early and for how long the flanker would be looked at. We observed that, overall, participants looked at the flanker only rarely and that whether participants looked at the flanker or not was modulated neither by target–distractor discriminability nor by congruency. This finding—that the flanker was not fixated in many trials and that participants completed the task without many fixations on the flanker—is novel as there is, to our knowledge, no study that assessed the effects of target–distractor discriminability in the flanker paradigm by measuring eye movements. The finding likely reflects that participants are effective at inhibiting eye movements to the flanker (see also [30]), irrespective of target–distractor discriminability or congruency. In a study [30] participants completed a visual search task and in Experiment 1B a distractor was always located at the top of the search array. Participants were made aware that the target would never appear at that location and hence discouraged from searching for the target in that location. The authors observed that fixations on the distractor were rare, in line with our results. Considering the strong flanker effect that was observed for the manual RT data in our study, it appears that the flanker effect may largely be a result of covert shifts of attention. Taking into account only the trials on which the flanker was looked at, we observed that participants looked at the flanker earlier in the high target–distractor discriminability than in the low target–distractor discriminability condition. Thus the results for fixations on the flanker and target may suggest that more difficult search is associated with the first few search moves being made to the search array itself (looking at the target later for low target–distractor discriminability) before the flanker is looked at (looking at the flanker later for low target–distractor discriminability). However, we would like to acknowledge that the results regarding fixations on the flanker should be interpreted with caution because the flanker was fixated in only a relatively small percentage of trials and further investigations are warranted. However, exactly this finding—few fixations on the flanker—adds the new knowledge that manual RT flanker effects are largely due to shifts of covert attention.

To summarize, the RT results of Experiment 2 replicated Experiment 1. The fixation data reflected the more difficult search process when targets and distractors share colour and are hard to discriminate, since there were more fixations until the target was reached with lower than higher target–distractor discriminability and more fixations until the flanker was looked at for low than high target–distractor discriminability trials. The analyses of fixations on the flanker revealed that the flanker was fixated only rarely and thus, considering the strong congruency effect in the RT data, it appears that the flanker effect is largely driven by covert allocation of attention to the flanker. For future work it would be of high interest to assess eye movements in a similar task also in a VR environment as it may be possible that saliency of targets may play a smaller role in attracting gaze in more natural environments [23] and that a smaller number of fixations may occur in 3D, possibly because eye movements are more costly in 3D than 2D [22].

## 4. Conclusion

The goals of our study were to accomplish a transfer of traditional paradigms used for assessing attention and distraction to an immersive VR environment and to explore the attentional effects when daily objects are being used as stimuli. We wished to take a first step in the direction of investigating distraction and response competition in a VR environment and to this end, we ensured contact with previous studies that have been carried out in the laboratory but at the same time increased ecological validity.

Our two experiments showed that participants took more time to indicate which one of two target objects was present in a search array when targets and distractors shared the feature colour and were harder to discriminate than in a condition in which colour is distinct. Further, the manual RT data also showed significant congruency effects, indicating that typical congruency effects can be observed during testing in VR and when using daily objects as stimuli. This indicates that the assessment of attentional effects that have usually been investigated in the laboratory, in particular the implementation of a flanker paradigm, is feasible in immersive VR, accomplishing the desired transfer. This conclusion is also supported by the replication of the effects observed in Experiment 1 by Experiment 2.

By using this technology and presenting daily items we made a significant step towards increasing ecological validity and we are hoping that our study will encourage the use of immersive VR for future studies. Here we used a combination of visual search and the flanker paradigm similar to previous research in order to make contact with the literature and the described effects. We employed VR as a medium for visual presentation of stimuli and the next important steps would include taking advantage of the more ecologically valid context that VR offers for studying cognition. We would like to suggest that future studies may wish to devise even more realistic settings and tasks, and could also include other response modalities than a simple button press. Assessments in immersive VR provide many options such as reaching, pointing and moving in the environment. Future work could thus make use of those exciting possibilities that cannot be applied using traditional methods. In addition, manipulating cognitive and motor demands, e.g. by asking participants to complete additional cognitive or motor tasks in VR, as is often the case in daily life (e.g. moving towards or grasping some object while searching for others) would be an interesting avenue to take. Further, the array of target objects could be extended to assess which features of everyday objects and also in how far semantic guidance may affect search performance. For instance, one study [23] showed that some objects required more fixations in order to be located than others.

Experiment 1 showed a correlation between age and the flanker effect for high target–distractor discriminability trials. While our analyses were of a rather explorative nature we would like to point out the importance of assessing effects of ageing on attention in VR (see [19]).

Taken together, VR offers a new and interdisciplinary perspective and a wealth of possibilities to investigate attention and distraction. We hope that our experiments will motivate such work.

## Footnotes

1 On some trials participants looked at the flanker more than once. Those instances are included in this analysis to assess dwell times.

## Data accessibility

Our data and code are deposited at the Dryad Digital Repository: https://doi.org/10.5061/dryad.9s85m06 [32]
